# Prevalence and associated factors of diarrhea among under-five children in Debre Berhan town, Ethiopia 2018: a cross sectional study

**DOI:** 10.1186/s12879-020-4905-3

**Published:** 2020-02-24

**Authors:** Sisay Shine, Sindew Muhamud, Solomon Adanew, Alebachew Demelash, Makda Abate

**Affiliations:** 1 Public Health Department, College of Health Science, Debre Berhan University, P.O.Box: 445, Debre Berhan, Ethiopia; 2 Nursing Department, College of Health Science, Debre Berhan University, Debre Berhan, Ethiopia; 3 Midwifery Department, College of Health Science, Debre Berhan University, Debre Berhan, Ethiopia

**Keywords:** Children, Diarrhea, Debre Berhan, Ethiopia

## Abstract

**Background:**

Diarrhea is responsible for 525,000 children under-five deaths and 1.7 billion cases globally and is the second leading cause of death among children under-five every year. It is a major public health problem in low income countries like Ethiopia. The main aim of this study was to assess the prevalence of diarrhea and associated risk factors among children under-five in Debre Berhan Town, Ethiopia.

**Methods:**

A community-based cross-sectional study was conducted in 420 parent or caretaker/children pairs in Debre Berhan town between 13 and 18 April 2018. A multi-stage sampling strategy was used to select the study participants. Data were collected using pre-tested and structured questionnaires. Data were entered in Epi-info computer software version 3.5.1 and exported to SPSS Window Version-16 for analysis. Adjusted odds ratio with 95% confidence intervals were used to assess the level of significance.

**Results:**

The two week prevalence of diarrhea among children under-five was 16.4% (69/351). Children aged 7–11 months (adjusted odds ratio (AOR): 4.2, 95% confidence interval (CI): 1.2–15.3), being the second-born child (AOR: 3.9, 95%CI: 1.8–8.5), not vaccinated against rotavirus (AOR: 10.3, 95%CI: 3.2–91.3) and feeding children by hand (AOR: 2.5, 95%CI: 1.1–6.1) were significant predictors of diarrhea.

**Conclusions:**

This study revealed that the two weeks period prevalence of diarrhea among children under-five years was 16.4%. Education programs on the importance of vaccination against rotavirus, increasing breast feeding frequency with complementary food after six months and the critical points of hand washing are recommended.

## Background

Diarrheal disease is a major public health problem worldwide. Globally, 525,000 children under-5 years die due to diarrhea every year, roughly 2195 every day [1]. This represents 8% of all deaths and is the second leading cause of death among children under-5 years old [2]. Annually, 1.7 billion diarrhea episodes occurred among children under-5 years worldwide [3]. The majority of morbidity and mortality occurred in south Asia and sub-Saharan African countries, which 88% were attributable to unsafe water, inadequate sanitation, and insufficient hygiene [4].

Despite global diarrheal deaths among children of under 5 years decreasing by 60% between 2000 and 2017. Ethiopian morbidity reports and community-based studies indicate that diarrheal diseases accounted for 20% of childhood death and 22% of childhood diarrheal disease in 2000 [3, 5, 6].

Studies in Ethiopia also showed that low maternal education, poor sanitation, contaminated water source, duration of breast feeding, failure to wash hands, absence of rotavirus vaccination, failure to dispose of feces hygienically, age of child and adequate food hygiene were significant predictors of diarrheal disease occurrence in children under-5 years [7–13].

However, information related to diarrheal disease in children under-five in Debre Berhan is limited. Therefore, the aim of this research was to assess the prevalence of diarrheal disease and associated factors in children under-5 years old in Debre Berhan town, Ethiopia. The findings will contribute to improving the lives of children under-5 years. In addition, the information can be used to develop effective educational program to improve child health overall. Similarly, this research will inform policy makers and program initiators on acceptable services.

## Methods

### Study area and period

The study was conducted in Debre Berhan town from April 13–28, 2018. Debre Berhan town is found in North Showa Zone of Amhara regional state, around 130 km away from the capital of Ethiopia, Addis Ababa. It has one referral hospital, 3 health centers, 14 clinics and around 16 pharmacies. Based on the Debre Berhan town health administration office report, the current population of the town is 103,450 of whom 46, 553 are men, 56,897 are women. From the total population, 14,011 are children under-5 years.

### Study design

A community-based cross-sectional study design was used to assess the prevalence of diarrhea and associated factors among children under-5 years in Debre Berhan town.

### Study population

All children under- 5 years old with their mothers or caretakers who live in selected kebeles of Debre Berhan town were our study population.

### Sample size determination

Sample size was determined based on the formula used to estimate single population proportion, assuming 21.5% 2 week period prevalence of diarrhea among children under-5 years [14], and a 5% margin of error with 95% confidence level. The sample size calculated was 256. After adjusting for a non-response rate of 10% and design effect of 1.5 the final sample size required was 428 mother/caretaker-child pairs.

### Sampling procedure

Multi-stage sampling was used to obtain a representative sample of the study participants. Firstly, four kebeles were selected from the total fourteen kebeles using a lottery method. Then, a census was conducted in each of the selected kebeles to identify eligible households. Finally, households were selected using systematic random sampling with a mother/caretaker-child pair selected from each household until the required sample size was fulfilled.

### Operational definitions

#### Diarrhea

The passage of three or more loose or watery stool in a 24 h period, as reported by the mother/caretaker of the child [4].

#### Caretaker

Any person who provides care for the child other than the mother.

### Data collection tool and methods

Data were collected by seven trained midwives and three supervisors. Structured questionnaires were developed in English after review of different literature and guidelines. English version questionnaires were translated into Amharic language and again translated back into English by experts fluent in both languages (Additional file 1). The training was given for data collectors and supervisors for 2 days on the study instrument and data collection procedures. Pre-testing was conducted on 5% of the total sample size. Data were collected by face to face interview.

### Data processing and analysis

The data collected from the field were edited, checked for completeness and consistency, coded and entered into Epi-info computer software version 3.5.1. Once entered, the data were exported to SPSS Window Version-16 for cleaning and further analysis. Both descriptive and inferential statistics were employed in the analysis. Bivariate logistic regression analysis was performed for each independent variable with the outcome variable and those variables with a *p*-value of < 0.2 included in multivariable logistic regression analysis to identify predictors of diarrhea. Enter method was used to select the variables in multivariate logistic regression analysis. Multicollinearity test was performed to assess the existence of correlation among the predictor variables. Additionally, goodness off fit to the final model was checked by Hosmer and Lemeshow and was found fit. Adjusted odds ratios with 95% confidence intervals were calculated and *P*-values less than 0.05 were considered statistically significant.

## Results

### Socio-demographic characteristics of the mother/caretaker and children

A total of 420 mother/caretaker completed the questionnaires. The majority 50.2% (211/420) of the mothers/caretakers were 20–29 years of age with a mean age of 29.7 years and SD of + 4.6 years. From the total study participants, 86.7% (364/420) were married and 83.6% (351/420) were orthodox religion. The majority 70.0% (294/420) of the household family size were between 1 and 4 people. Among the total respondents, 31.9% (134/420) had a college education or additional qualification. More than half 55.5% (233/420) of the children were male and 21.2% (89/420) were 12–23 months of age (Table 1).

**Table 1:** Socio-demographic characteristics of mother/caretaker and children in Debre Berhan Town, Ethiopia 2018

Variable	Frequency	Percent (%)
Relation of the respondent to the child		
Mother	387	92.1
Care taker	33	7.9
Age of mother/caretaker		
< 20	13	3.1
20–29	211	50.2
30–39	175	41.7
> =40	21	5.0
Sex of children		
Male	233	55.5
Female	187	44.5
Age of children (in months)		
0–6	59	14.0
7–11	46	11.0
12–23	89	21.1
24–35	75	17.9
36–47	81	19.3
48–59	70	16.7
Educational status of mother/care taker		
Can’t read and write	43	10.2
1–4 grade	42	10.0
5–8 grade	81	19.3
9–12 grade	120	28.6
College and above	134	31.9
Family size of the household		
1–4	294	70.0
> =5	126	30.0
Household monthly income (Ethiopian Birr)		
< =3438	247	58.8
> 3438	173	41.2

### Environmental and hygiene characteristics

In the study area, all of households have latrines in their dwellings. The majority 45.0% (189/420) of the households have traditional pit latrines and 61.9% (260/420) dispose of the solid waste by private vendors. The majority 80.2% (337/420) dispose of children’s feces in the toilet. A further 57.6% (242/420) dispose of household waste in the seepage pits and 80.2% (337/420) of households get their water from a pipe (Table 2).

**Table 2:** Environmental characteristics of household in Debre Berhan town, Ethiopia, 2018

Variable	Frequency	Percent
Type of latrine used by households		
Traditional pit latrine	189	45.0
Ventilated improved pit latrine	107	25.5
Shared latrine	84	20.0
Flush latrine	40	9.5
Solid waste disposal methods		
Privately prepared pit hole	20	4.8
Burning	93	22.1
Collected by private vendors	260	61.9
Dumped in street	47	11.2
Liquid waste disposal methods		
Septic tank	54	12.9
Seepage pit	242	57.6
Open surface	124	29.5
Children’s feces disposal methods		
Toilet	337	80.2
Covered by soil	49	11.7
Open space	34	8.1
Source of water		
Unprotected spring	40	9.5
Piped	337	80.2
Protected spring	43	10.3
Distance of water source to home		
< 15 min	388	92.4
> =15 min	32	7.6
Hand washing facility beside toilets		
Yes	178	42.4
No	242	57.6

### Health and dietary characteristics of children

From the study participants, 50.0% (210/420) of children under-five were the first-born of their family. About, 90.7% (381/420) of the children were born in health institutions. The majority 71.2% (299/420) of children under-five started complementary feeding at 6 months of age. About, 79.5% (334/420) of children were vaccinated against measles and 96.0% (403/420) against rotavirus (Table 3).

**Table 3:** Health and dietary characteristics of children under-five in Debre Berhan Town, Ethiopia 2018

Variable	Frequency	Percent
Birth order		
First	210	50.0
Second	121	28.8
Third	49	11.7
Fourth and above	40	9.5
Place of delivery		
Home	39	9.3
Health institution	381	90.7
Duration of breast feeding		
0–23 months	244	59.8
24 months	61	15.0
> =25	103	25.2
Got rotavirus vaccination		
Yes	403	96.0
No	17	4.0
Got measles vaccination		
Yes	334	79.5
No	86	20.5
Complementary food started		
At 6 month	299	71.2
Before 6 month	33	7.9
After six month	88	20.9

### Prevalence of diarrhea among children under-five years

The prevalence of diarrhea among children under-five was reported to be 16.4% [95%CI: 12.7–20.0] in the 2 week surveillance period, of whom 8.6% (36/420) were males. Among children who had diarrhea, 15.9% (67/420) experienced watery diarrhea with a few reporting bloody or mucoid stools. The prevalence of diarrhea was 2.6% (11/420) in children aged 7–11 months (Table 4).

**Table 4:** Bivariate and multivariate analysis on determinants of under-five diarrhea in Debre Berhan Referral Hospital, Ethiopia 2018

Variables	Diarrheal status of child		COR (95%CI)	AOR (95%CI)
	Yes	No		
Age of children (in months)				
0–6	6 (1.4)	53 (12.6)	1.2 (0.6–8.2)	0.3 (0.1–16.2)
7–11	11 (2.6)	35 (8.3)	1.9 (0.7–5.3)	4.2 (1.2–15.3)*
12–23	16 (3.8)	73 (17.4)	1.3 (0.8–6.1)	0.9 (0.3–3.0)
24–35	15 (3.6)	60 (14.3)	1.5 (0.5–4.0)	1.1 (0.3–3.5)
36–47	11 (2.6)	70 (16.7)	0.9 (0.5–4.3)	0.4 (0.2–1.7)
48–59	10 (2.4)	60 (14.3)	1.0	1.0
Birth order of children				
First	28 (6.7)	182 (43.3)	1.0	1.0
Second	28 (6.7)	93 (22.1)	2.0 (1.2–3.1)	3.9 (1.8–8.5)*
Third	3 (0.7)	46 (11.0)	0.4 (0.2–2.1)	0.2 (0.1–1.3)
Fourth and above	10 (2.3)	30 (7.2)	2.2 (0.1–4.8)	1.4 (0.4–5.1)
Weaning age of children				
At six month	50 (11.9)	249 (59.3)	1.0	1.0
Less than six month	10 (2.4)	43 (10.2)	1.2 (0.2–2.2)	0.6 (0.1–1.8)
Above six month	9 (2.1)	59 (14.1)	0.8 (0.1–1.1)	0.2 (0.1–1.4)
Rotavirus vaccination				
Yes	62 (14.7)	341 (81.2)	1.0	1.0
No	7 (1.7)	10 (2.4)	3.6 (1.4–10.5)	10.3 (3.2–91.3)*
Feeding child by using hand				
Yes	32 (7.6)	133 (31.7)	1.4 (1.2–3.1)	2.5 (1.1–6.1)*
No	37 (8.8)	218 (51.9)	1.0	1.0

*Significant at P < 0.05

### Factors associated with diarrhea among children under-five years

In the bivariate analysis, age of child, birth order, age of starting complementary food, vaccination against rotavirus and feeding children by hand were significantly associated with diarrhea (Table 4). The results from multivariate logistic regression analysis revealed the odds of developing diarrhea among children in the age group 7–11 months were 4.2 times higher (AOR: 4.2, 95%CI: 1.2–15.3) compared to the 48–59 month age group. Being the first-born (AOR: 3.9, 95%CI: 1.8–8.5), unvaccinated against rotavirus (AOR: 10.3, 95%CI: 3.2–91.3) and hand-feeding of child (AOR: 2.5, 95CI: 1.1–6.1) were significant predictors of diarrhea (Table 4).

## Discussion

This study was conducted to assess the prevalence and associated factors of diarrhea among children under-five in Debre Berhan town, Ethiopia. The Ethiopian Demographic and Health Surveys of 2016, showed that diarrheal disease was the leading cause of illness among children under-5 years.

The result of this study showed that prevalence of diarrhea among children under-five was 16.4% (95%CI: 12.7–20.0). This finding was congruent with the study done in Dale District, Sidama zone, Southern Ethiopia, 13.9% [11], Yaya Gulele district, Ethiopia 13.5% [15], Serbo town, Southwest, Ethiopia, 14.9% [16], Bahr Dar city, 14.5% [17], and Farta Wereda, Northwest Ethiopia, 16.7% [7]. However, result of this study was lower than the study conducted in Sena’a, Yemen, 29.07% [18], Senegal, 26% [19], Cameroon, 26.1% [20], Sheka zone, southwest Ethiopia, 21.8% [12], Jig-Jiga city, Eastern Ethiopia, 27.3% [21], Bahir Dar Zuria district, Northwest Ethiopia, 20% [22], North Gondar zone, 21.1% [10] and Harena Buluk district, Southeast Ethiopia, 28.4% [23]. In contrast, it was higher than the study conducted in Wolayta Sodo town, Southern Ethiopia, 11.0% [24]. This difference may be due to seasonal trends in diarrhea disease or differences in year and age of the study participants as well as the differences in the study design and data collection.

Children aged between 7 and 11 months were at high risk of developing diarrhea compared with children aged was less than 7 months. This result was in line with the results of the study conducted in Farta Wereda, Northwest Ethiopia [7]. The increased risk might be due to the decline/loss in maternal antibodies and at this age child start complementary feeding that might increase their exposure to contaminated foods and water. In addition, crawling begins at this age further increasing potential exposure to fecally contaminated environments.

This study found that diarrhea was more common among second-born children compared with first-born children (AOR: 3.9, 95%CI: 1.7–8.5). Similarly, a cross-sectional study conducted in Jig-Jiga district, Somali region, Ethiopia showed that fourth-born children and above were more affected by diarrhea compared with first borne [21]. This may be due to quality of care and attention from parents decreases as mothers become incapable of caring for children [25].

According rotavirus vaccination were 10.3 times more likely to have diarrhea compared with children who received rotavirus vaccination (AOR: 10.3, 95%CI: 1.2–91.2). The result suggests that a major contributor to the diarrheal burden in children less than 5 years in the town is in fact rotavirus. This result was in agreement with study done in Farta Woreda, Northwest Ethiopia [7].

In this study, the following limitations were noted. The major limitation of the study was the limited time period over which the study was conducted, that may create over or under reporting of diarrhea since diarrheal diseases have some seasonal variations.

## Conclusions

The 2 week prevalence of diarrhea among children under-5 years in Debre Berhan town was 16.4%. Childhood diarrheal disease was significantly associated with the age of children, birth order and hand feeding practice of mother. So, education program on the importance of vaccination against rotavirus, increase breast feeding frequency with complementary food after 6 months and the critical points of hand washing are recommended.

## Supplementary information

**Additional file 1.** Questionnaire on Diarrheal study

## Data Availability

The datasets used and/or analyzed during the current study available from the corresponding authors on reasonable request.

## Abbreviations

AOR: Adjusted odd ratio
BSc: Bachelors of science
CI: Confidence interval
SD: Standard deviation
