# Supplementary material for: Prevalence and associated factors of diarrhea among under-five children in Debre Berhan town, Ethiopia 2018: a cross sectional study
Source: BMC Infect Dis. 2020 Feb 24;20:174. doi: 10.1186/s12879-020-4905-3 (PMC7041267; doi:10.1186/s12879-020-4905-3)
Supplement: Supplementary file 1 — Additional file 1. Questionnaire on Diarrheal study [file 12879_2020_4905_MOESM1_ESM.docx]

## Annex III: Questionnaires in English version

Questionnaire code: ____________

**PART ONE: Socio demographic and Socio economic factors**

| **NO** | **QUESTIONS** | **RESPONSE** | **CODE** |
| --- | --- | --- | --- |
| 1.1 | What is the relation of the respondent to the child? | Mother | 1 |
|  |  | care taker | 2 |
| 1.2 | What is the Age of the mother or care taker? | -------------- |  |
| 1.3 | What is the marital status of the mother or care taker? | Married | 1 |
|  |  | Divorced | 2 |
|  |  | Single | 3 |
|  |  | Widowed | 4 |
| 1.4 | What is the religion of the mother or care taker? | Orthodox | 1 |
|  |  | Muslim | 2 |
|  |  | Protestant | 3 |
|  |  | Catholic | 4 |
| 1.5 | What is the educational status of the mother or care taker? | Can’t read and write | 1 |
|  |  | 1-4 | 2 |
|  |  | 5-8 | 3 |
|  |  | 9-12 | 4 |
|  |  | College and above | 5 |
| 1.6 | What is the occupation of the mother or care taker? | Government employee | 1 |
|  |  | Self-employee | 2 |
|  |  | House wife | 3 |
|  |  | Merchant | 4 |
|  |  | Farmer | 5 |
| 1.7 | Family size | __________in number |  |
| 1.9 | Average monthly income of the family | __________ETB |  |

**PART TWO: ENVIRONMENTAL AND HYGIENE CONDITION**

| **NO** | **QUESTIONS** | **RESPONSE** | **Code** |
| --- | --- | --- | --- |
| 2.1 | Is latrine available in your home? | Yes | 1 |
|  |  | No | 2 |
| 2.2 | What kind of toilet facility do members of household usually use? | Flush connected to municipal sewer line | 1 |
|  |  | Flush connected to septic tank | 2 |
|  |  | Ventilated improved pit latrine | 3 |
|  |  | Pit latrine made from concrete slab | 4 |
|  |  | Shared latrine | 5 |
| 2.3 | How do you dispose household solid wastes or refuse? | In a privately prepared pit – hole | 1 |
|  |  | In refuse pit collected by municipality | 2 |
|  |  | Dumped in street/open space | 3 |
|  |  | Burn | 4 |
|  |  | Garbage can | 5 |
|  |  | Collected by private establishment | 6 |
| 2.4 | Where do you dispose your household liquid waste? | In septic tank/latrine pit | 1 |
|  |  | In seepage pit | 2 |
|  |  | Anywhere in open space | 3 |
| 2.5 | What is your source of water for drinking? | Pipe | 1 |
|  |  | protected wall spring | 2 |
|  |  | unprotected wall spring | 3 |
| 2.6 | Distance of water source to home | ……….minutes/meter |  |
| 2.7 | Is there any hand washing facility beside the toilet? | Yes | 1 |
|  |  | No | 2 |
| 2.8 | Where do you dispose your under five child waste? | In the toilet | 1 |
|  |  | Left it open everywhere | 2 |
|  |  | covered by soil | 3 |

**PART THREE: INFORMATION ON THE STUDY CHILD**

| **NO** | **QUESTIONS** | **RESPONSES** | **CODE** |
| --- | --- | --- | --- |
| 3.1 | What is the age of your child? | …month |  |
| 3.2 | What is the sex of your child? | Male | 1 |
|  |  | Female | 2 |
| 3.3 | What is the Birth order of your child? | First | 1 |
|  |  | Second | 2 |
|  |  | Third | 3 |
|  |  | Fourth and above | 4 |
| 3.4 | Where did the child born? | Home | 1 |
|  |  | Health institution | 2 |
| 3.5 | Have you ever breast feed your child? | Yes | 1 |
|  |  | No | 2 |
| 3.6 | If your answer is yes for how long you breast feed your child? | ………..months |  |
| 3.7 | What is the current breast feeding status of the child? | Exclusive breast feeding | 1 |
|  |  | partial breast feeding | 2 |
|  |  | Not breast feeding | 3 |
| 3.8 | At what age the child started supplementary /weaning feeding? | ___________________month |  |
| 3.9 | Did the child receive  Measles vaccination? (For children’s  greater than nine months OF AGE) | Yes  -By the response of the respondents  -By checking the card | 1 |
|  |  | No | 2 |
| 3.10 | Did the child receive Rotavirus vaccination? | Yes (Rvv1, Rvv2)  -By the response of respondents  -By checking the card | 1 |
|  |  | No | 2 |
| 3.11 | Does your child experience diarrhea (three or more loose or watery stool in a twenty-four hour period) in the last two weeks (in the last 14 days)? | Yes | 1 |
|  |  | No | 2 |
| 3.15 | What actions do you take to stop the diarrhea from the child? | Take him/her to the health institution | 1 |
|  |  | Take him/her to the traditional healers | 2 |
|  |  | Give him/her ORS | 3 |
|  |  | Increase feeding | 4 |
|  |  | Decrease/stop feeding | 5 |
|  |  | Homemade treatment | 6 |
